# 

**Published:** 1887-12

**Authors:** 


					﻿Entered at the Post Office, at Austin, Texas, as Second Class Mall Matter.
Vol. III
DECEMBER, 1887.
No. 6.l
DANIEL'S
MEDICAL
JOURNA;L
F. E. DANIEL., M. D., Editor and Publisher, AUSTIN, TEXAS.
Northern Office, 1217 Filbert Street. Philadelphia.
Eugene Von Boekmann, Stream Book and Job Printer, Austin, Texas.
				

